# Influence of oxytocin receptor single nucleotide sequence variants on contractility of human myometrium: an in vitro functional study

**DOI:** 10.1186/s12881-019-0894-8

**Published:** 2019-11-12

**Authors:** F. Füeg, S. Santos, C. Haslinger, B. Stoiber, L. Schäffer, E. Grünblatt, R. Zimmermann, A. P. Simões-Wüst

**Affiliations:** 1 Department of Obstetrics, University Hospital Zurich, Schmelzbergstrasse 12/PF 125, 8091 Zurich, Switzerland; 2 Department of Child and Adolescent Psychiatry and Psychotherapy, University Hospital of Psychiatry Zurich, University of Zurich, Zurich, Switzerland; 3 Neuroscience Centre Zurich, University of Zurich and ETH Zurich, Zurich, Switzerland

**Keywords:** Oxytocin, Receptor, Sequence variants, Myometrium, Contractility

## Abstract

**Background:**

Oxytocin receptor (*OXTR*) gene variants have been shown to affect the prevalence of preterm birth, mode of delivery and oxytocin (OXT) requirements for labor induction and augmentation. We hypothesized that this might be associated with different myometrium responses to oxytocin. Our aim was to investigate the influence of a selection of eight *OXTR* gene single nucleotide variants on oxytocin-induced stimulation of human myometrium contractility in vitro.

**Methods:**

Human myometrium biopsies were collected during elective cesarean sections at term, if patients had given informed consent. Myometrial strips were submerged under tension in an organ bath and allowed to contract; the remaining material was stored at − 80 °C for further determination of relevant genetics and mRNA level. The area under the curve (AUC) of all contractions taking place in the absence of OXT and of those occurring upon OXT addition (for 30 min each) was measured. OXT stimulation, defined as the ratio between AUC measurements after OXT addition and those in the absence of OXT was calculated for each strip. TaqMan™ Assays were used to detect the allele distribution of the eight *OXTR* variants and to determine the relative amounts of OXTR-mRNA in the samples. For each variant, oxytocin stimulation of contractility was compared between samples homozygous for the reference allele (reference group) and samples with at least one variant allele (variant group) by linear regression.

**Results:**

Sixty samples were included in the present study. For rs1042778, rs11706648, rs4686301, rs53576, rs237895, and rs237902, OXT stimulation was similar in the reference and in the variant groups. However, the values of OXT stimulation differed significantly between the reference and the variant groups for rs4686302 (3.1 vs. 4.1 times; *p* = 0.022) and rs237888 (3.2 vs. 5.5 times; *p* = 0.001). No significant differences between the levels of OXTR-mRNA in the various reference and corresponding variant groups were detected.

**Conclusions:**

Patients with variant alleles of rs237888 and/or rs4686302 may be more sensitive to oxytocin stimulation, explaining why these sequence variants have been associated with lower cesarean section prevalence and premature birth, respectively.

## Background

Oxytocin (OXT) is a peptide of nine amino acids that exerts various effects in the human body, triggering both central and peripheral receptors (see e.g. [1–4]). Endogenous OXT plays important roles in the labor process, lactation and maternal behavior after birth. During labor, uterus contractions are triggered through the binding of endogenous OXT to its receptor in myometrium cells. If required, uterine activity can be stimulated with synthetic OXT [5] to induce or augment labor, to support placental delivery or to prevent postpartum hemorrhage. Towards the end of pregnancy [6] and during treatment with synthetic OXT [7], expression of oxytocin receptor (OXTR) in myometrium cells increases. Changes in OXTR expression, but also the existence of genetically variant forms of this receptor are likely to affect sensitivity towards OXT (and other OXTR binding medications), and to contribute to the variety of patient responses that are observed in clinical practice.

Previous experimental work has shown that a few rare single-nucleotide variants of *OXTR* might affect the binding of OXT to this seven-transmembrane-helix receptor and possibly affect OXT-mediated signal transduction [8]. Moreover, an association between a few common substitution OXTR variants and the prevalence of preterm birth has been suggested [8]. Other studies revealed that a combination of three common minor allelic variants from the *OXTR* can increase preterm birth risk [9], and that the minor allele of the common rs53576 variant is associated with a long transition to active labor [10]. Finally, there are some indications that *OXTR*single-nucleotide variants may affect the response to OXT-treatment with regard to labor induction and augmentation, mode of delivery and prevalence of preterm birth [11].

We hypothesized that at least some of the influences of *OXTR* variants on clinically-relevant outcomes occur through changes in OXT-mediated cell signaling. Our objective was to investigate whether some common *OXTR*single-nucleotide sequence variants that have previously been shown to affect perinatal outcomes are associated with different oxytocin-induced stimulation of human myometrium contractility in vitro.

## Methods

### Samples

In two original in vitro studies performed at the University Hospital of Zurich (see [12, 13]), the effects of various tocolytic agents on term myometrium contractility were compared. In these studies, contractility of myometrium strips was often measured in the absence and/or in the presence of OXT, and, when enough material was available, tissue samples were shock frozen with liquid nitrogen and stored at − 80 °C for further determination of relevant genetics and mRNA level. Patients were asked to donate a myometrium sample if they were going to undergo an elective cesarean section at term, had a singleton pregnancy, had not received tocolytics during the last 2 weeks, had had no previous cesarean section, had no premature rupture of membranes, had no amniotic infection syndrome, and had no preeclampsia or HIV-Infection. During surgery, a myometrium sample of about 5 g was removed from the patient’s uterus by the physician in charge and placed immediately in ice-cold Ringer’s solution. Part of the sample was further processed for the contractility experiments, the remaining material was cut in pieces of approximately 60 mg, shock frozen in liquid nitrogen and kept at − 80 °C until DNA and RNA extraction*.* For the present study, samples were included if contractility data in both the absence and presence of OXT to stimulate contractions of maximal strength (either 1 U/mL or 40 U/mL) was available for at least one strip and enough DNA and RNA could be extracted. These conditions were fulfilled by 60 out of 133 possible samples.

### Oxytocin-stimulated myometrium contractility

Part of the myometrium sample was cut in longitudinal strips of about 15 × 2 × 1 mm. These strips were mounted on the myograph (Myograph DMT800MS, Muscle Strip Myograph System, DMT, Denmark; provided by ADInstruments GmbH, Spechbach, Germany) between two clamps in a chamber filled with 6 mL of Krebs solution (118 mM NaCl, 24.9 mM NaHCO_3_, 4.7 mM KCl, 1.24 mM KH_2_PO_4_, 2.48 mM CaCl_2_, 1.21 mM MgSO_4_, 10 mM glucose, 0.034 mM EDTA, pH = 7.4). The temperature was regulated to 37 °C and the medium was enriched with a gas mixture (95% O_2_ and 5% CO_2_, Pan Gas, Dagmarsellen, Switzerland). Contraction data was transferred to a PC for analysis with the software Labchart Pro 8.0.6 (ADInstruments GmbH, Spechbach, Germany) via a transducer (PowerLab 4/30, ADInstruments GmbH, Spechbach, Germany). The strength (measured in grams, g) of regular spontaneous myometrium contractions (i.e. in the absence of OXT) and of contractions occurring upon OXT addition were recorded for 30 min each. The area under the curve (AUC, g x s) of all contractions taking place in the absence of OXT and of all contractions occurring upon OXT addition (for 30 min each) was measured and taken as an indicator of contractility. OXT stimulation, defined as the ratio between AUC measurements after OXT addition and those in the absence of OXT was calculated for each strip. OXT was used at concentrations known to maximally stimulate contractility in vitro (1 and 40 U/mL, depending on the original study).

### DNA and RNA extraction

All protocols were performed according to the manufacturers’ instructions. Frozen myometrium tissue was removed from the − 80 °C freezer and placed immediately in liquid nitrogen to prevent tissue thawing. A small piece of frozen myometrium tissue (ca. 60 mg) was cut and ground to a fine powder by using a cold mortar and pestle. For at least 2 h before starting this procedure, the mortar and pestle were stored at − 80 °C; all instruments and 1.5 mL Eppendorf tubes used were placed in liquid nitrogen before starting. While the myometrium tissue was being ground, small amounts of liquid nitrogen were added to prevent thawing of the tissue. About 40 mg of the ground powder was transferred to a 1.5 mL Eppendorf tube that was in the liquid nitrogen. The tube with the powder was placed in liquid nitrogen until the other samples were prepared. The remaining tissue was stored again at − 80 °C. DNA and RNA were extracted with the AllPrep DNA/RNA/Protein Kit (Qiagen) and were quantified using a Nanodrop™1000 (Thermo Scientific, USA).

### Determination of a selection of *OXTR* single-nucleotide sequence variants

TaqMan™ Assays (Applied Biosystems, Forster City, CA, USA) were used to detect the allele distribution of eight common *OXTR*single-nucleotide sequence variants previously shown to be associated with a relevant perinatal outcome (see additional annotations and functional data extracted from SNPnexus http://www.snp-nexus.org/ in Additional file 1: Table S1). Genotyping was performed on DNA extracted as described above, using 384 well plates sealed with an optic adhesive film in a ViiA 7 Real-Time PCR System (Applied Biosystem, Forster City, CA, USA). The investigated variants (and assays ID) were: rs1042778 (C__7622140_30), rs11706648 (C__3290321_10), rs237888 (C__3290324_10), rs4686301 (C__3290326_10), rs53576 (C__3290335_20), rs237895 (C__1750495_10), rs237902 (C__3290345_30) and rs4686302 (C__3290346_20).

### Quantification of mRNA

To compare the relative amounts of *OXTR*-mRNA in the various myometrium samples, cDNA was first synthesized from RNA (QuantiTec® reverse transcription Kit, Qiagen, CA, USA) and subsequently submitted to quantitative real-time PCR (ViiA 7 Real-Time PCR System; Applied Biosystems, Forster City, CA, USA). The cDNA corresponding to the *OXTR*-mRNA (assay Hs00168573_m1), as well as to the reference genes *GAPDH*-mRNA (Hs02786624_g1), 18S rRNA (Hs03003631_g1) and *EEF1A1*-mRNA (Hs00265885_g1) were determined. Cycle threshold (C_T_) values were determined and mean PCR efficiencies were calculated using the ‘LinRegPCR’ program [14]; the latter were in a range between 80 and 95%. Quantification of *OXTR-*mRNA levels normalized to *GAPDH*-mRNA, 18S rRNA and *EEF1A1*-mRNA was carried out using ‘Biogazelle qBASE plus’ software (Biogazelle, Zwijnaarde, Belgium) [15], thus taking into account the specific PCR efficiency for each reaction. This software enables normalization to more than one reference gene, is able to take into account gene-specific PCR efficiency and also employs signals from inter-run calibration to minimize run-to-run variation, which distinguishes it from the ΔCt method. The data are expressed as Calibrated Normalized Relative Quantities (CNRQ).

### Data analysis

Throughout this manuscript the genetic nomenclature according to recommendations of Human Genome Variation Society (HGVS) and American College of Medical Genetics and Genomics (ACMGG) is used [16, 17].

For each sequence variant, samples were divided into two groups according to genotype; i.e. whether (1) they were homozygous for the reference allele (reference group) or (2) possessed at least one variant allele (variant group). In each case, reference and variant group were compared by linear regression. The OXT concentration used in the two original studies was considered a potential covariate in the association between reference group and variant group; confounding was evaluated by adding this variable to the linear regression model and looking at the change of the regression coefficients. Data was analyzed with IBM® SPSS® Statistics Version 23 and is shown as mean ± S.D., unless otherwise indicated. Two-tailed*p*-values < 0.05 were considered statistically significant. Link disequilibrium (LD) was determined using LDlink 3.0. (https://ldlink.nci.nih.gov/; see Additional file 2: Table S2 for the LD of all variants studied). Hardy-Weinberg Equilibrium was tested for each of the variants using gene calculator https://www.genecalculators.net/associatorrr-cc.html. *P*-value < 0.05 indicated disequilibrium of the genotypes (results see Additional file 3: Table S3).

## Results

As was to be expected from the criterion of including samples collected during the two original studies, all myometrium samples considered in the present analysis (*n* = 60) were capable of contracting spontaneously in vitro and were able to contract long enough for spontaneous contractility characterization as well as for contractility measurement upon addition of OXT to the myograph chamber. Therefore, OXT stimulation could be calculated in all cases. Its average was 3.4 times the value of the initial, spontaneous contractility (S.D. 1.5, median 3.0, minimum 0.8, and maximum 10.5). The main characteristics of the patients who donated the myometrium biopsies used in the present functional study are summarized in Table 1.

**Table 1:** Characteristics of the patients who donated the myometrium biopsies analyzed in the present work (*n* = 60)

Characteristic	Values ^a^
Age (years)	34.0 ± 5.1
BMI (before pregnancy)	26.1 ± 8.5^b^
Gravidity (number)	2.0 ± 1.2
Parity (number)	1.4 ± 0.6
Comorbidities	
hypothyroidism	10
gestational diabetes	10^c^
obesity (BMI ≥ 30)	6
overweight (BMI ≥ 25)	3
gestational hypertension	3
hyperthyroidism	1
uterine atony	1
osteoporosis	1
anorexia	1
Gestational age (weeks)	38.3 ± 0.7
Cesarean (number)	1.0 ± 0.0

^a^ for continuous variables given as mean ± S.D., otherwise as number

^b^ data available for *n* = 26

^c^ of these, 2 being treated with insulin

In Table 2, the selected *OXTR*single-nucleotide sequence variants are briefly described, together with their minor allele frequency (MAF)-values in the currently studied population, their MAF-values for the European population and previously described influence on perinatal outcomes (for further information on the variants see Additional file 1: Table S1). The number of samples with each one of the three possible allele combinations of each single-nucleotide variant are depicted in Additional file 3: Table S3. Given the very low number of samples homozygous for the variant alleles, samples were divided in two categories only for further calculations on each variant: the reference group, containing all samples with no variant allele and the variant group, in which all samples had at least one variant allele.

**Table 2:** Overview of the single-nucleotide sequence variants considered in the present work

Variant	Variant-type	Position (Ch37) ^a^	MAF (Eur.) ^b^	MAF (study) ^c^	Genotype (REF/MA)	Association
rs1042778	3’UTR	g.8794545	0.38	0.41	(G/T)	maximal oxytocin infusion rate / total oxytocin dose [11]
rs11706648	Intron	g.8796547	0.32	0.28	(A/C)	maximal oxytocin infusion rate [11]
rs237888	Intron	g.8797095	0.06	0.05	(T/C)	cesarean section [11]
rs4686301	Intron	g.8798586	0.31	0.26	(C/T)	maximal oxytocin infusion rate / total oxytocin dose [11]
rs53576	Intron3	g.8804371	0.35	0.34	(G/A)	time from latent to active labor [10]
rs237895	Intron	g.8807423	0.41	0.42	(C/T)	maximal oxytocin infusion rate [11]
rs237902	Exon 3	g.8809184	0.33	0.32	(G/A)	preterm birth (suggestive) [8]
rs4686302	Exon 3	g.8809222	0.12	0.13	(C/T)	preterm birth [8]

*MAF* Minor allele frequency

*REF/MA* Reference /Minor Allele gene

^a^ position according to the genomic reference sequence NCBI Build 37.3 GRCh37.p5 assembly

^b^ in the European population (all available EUR subgroups) according to the 1000 Genomes EUR population (SNPnexus http://snp-nexus.org)

^c^ in the present study population

OXT stimulation of myometrium contractility in the reference and variant allele groups of the selected *OXTR* sequence variants were compared (see Table 3). In most cases, OXT stimulation was similar in the two groups compared. However, the values of OXT stimulation differed significantly between the reference and the variant groups for rs4686302 (3.1 vs. 4.1 times; *p* = 0.022) and rs237888 (3.2 vs. 5.5 times; *p* = 0.001).

**Table 3:** Oxytocin stimulation^a^ of myometrium contractility in the various reference and variant allele groups

	Oxytocin stimulation (AUC after OXT addition/ AUC spontaneous contractility)				
	Reference group		Variant group		*p*-value^a^
	n/N	Mean ± SD	n/N	Mean ± SD	
rs1042778	21/60	3.0 ± 1.3	39/60	3.5 ± 1.6	0.192
rs11706648	29/60	3.5 ± 1.7	31/60	3.2 ± 1.3	0.435
rs237888	54/59^b^	3.2 ± 1.2	5/59	5.5 ± 2.9	**0.001**^**c**^
rs4686301	32/60	3.4 ± 1.7	28/60	3.3 ± 1.4	0.697
rs53576	25/60	3.3 ± 0.9	35/60	3.4 ± 1.9	0.974
rs237895	18/60	3.4 ± 1.0	42/60	3.3 ± 1.7	0.843
rs237902	26/60	3.8 ± 1.8	34/60	3.3 ± 1.3	0.939
rs4686302	45/60	3.1 ± 1.3	15/60	4.1 ± 2.0	**0.022**^**c**^

*n* Number of samples either in the reference group or in the variant allele group of a given single-nucleotide sequence variant

*N* Number of samples for which data on a given variant are available

*SD* Standard deviation

^a^ For each variant, the p-value represents the statistical significance of the difference between reference and variant groups as calculated using linear regression

^b^ One sample, in which the result of the allelic determination plot was undetermined, was excluded

^c^ Statistically significant

During the original studies performed on myometrial contractility, two different concentrations of OXT were used (1 U/mL or 40 U/mL) to stimulate contractions of maximal strength. To verify whether these different concentrations had an impact on the results, OXT concentration was added as a co-variate to the linear regression model used to compare reference and variant groups (multivariate model). Comparison of the beta coefficients with those obtained in the univariate linear regression model showed that, both in the case of rs237888 and of rs4686302, the differences between reference and variant groups remained significant (*p* = 0.001 and *p* = 0.028, respectively).

We have considered the possibility that rs237888 and rs4686302 could co-exist in some samples. Only one sample had both allele variants rs237888 and rs4686302; interestingly, this was the sample with the highest OXT stimulation. To find out whether rs237888 and rs4686302 are in LD, a linkage disequilibrium analysis within the European population was performed. This analysis revealed that no disequilibrium between rs237888 and rs4686302 exists ((EUR population R^2^ = 0.003, D’ = 0.577; Additional file 2: Table S2). In some of the sequence variants studied, high LDs were detected, e.g. rs4686301 and rs11706648 (EUR population R^2^ = 0.884, D’ = 0.967), and low LD between rs237895 and rs53576 (EUR population R^2^ = 0.718, D’ = 0.962). However, most of the sequence variants studied had low to no LD (see Additional file 2: Table S2).

The relative levels of *OXTR*-mRNA varied among the study population. Since the expression level of *OXTR* is likely to affect myometrium contractility and sequence variants might affect *OXTR* expression, the levels of *OXTR*-mRNA in the various reference and variant groups were compared. For each case, no significant differences between the levels of *OXTR*-mRNA in the reference group and the variant group were detected (s. Additional file 4: Table S4).

## Discussion

### Main findings

Our data reveals that two *OXTR* sequence variants previously shown to be associated with birth-related outcomes – rs237888 and rs4686302 – significantly affect the OXT-induced stimulation of myometrium contractility in vitro. In other words, the increase in contraction force upon OXT stimulation in the groups of samples with these *OXTR* allele variants was higher relative to the reference groups. Other sequence variants previously shown to be associated with birth-related outcomes – rs1042778, rs11706648, rs4686301, rs53576, rs237895, and rs237902 – did not seem to affect OXT stimulation of contractility under our experimental conditions.

### Strengths and limitations

In this functional in vitro study, the effects on myometrium response to OXT were observed in an assay that closely reflects the biological environment. Moreover, the myograph-setup allows working under defined conditions. These aspects can be seen as major strengths of our study, which complements well previous work based on clinical and epidemiological data. There were also some limitations, because the data on contractility resulted from pooling two previous original studies designed to address other research questions. These original studies comprised measurements of contraction strength in the absence and in the presence of only one oxytocin concentration (dose-dependency experiments may have been more appropriate for the present research question) in a moderate number of samples.

### Interpretation

The subgroup with at least one variant allele at rs237888 (T) showed a higher in vitro OXT stimulation of contractility than the corresponding reference subgroup. A previous study showed an association between this sequence variant and a lower risk for cesarean section among women induced near term. This lower cesarean section rate was associated with a lower OXT dose, lower maximal OXT infusion rate and lower duration of labor induction with OXT [11]. Increased response of the myometrium to OXT stimulation might explain the observed lower cesarean rate. However, it is still unclear, how this association can be explained in detail in terms of molecular/cellular biology. The variant allele at rs237888 is located in an intron, i.e. has no effect on the protein-coding sequence. Moreover, no effect on expression was observed (levels of mRNA were comparable between variant allele and reference subgroups). A search of the GTEx database for the impact of this variant on the expression of *OXTR* was also completed, but yielded no results (www.gtexportal.org/home, 28.09.2018, expression quantitative trait loci, eQTL). However, a splice variant located at rs237888 as well as at the transcription factor binding sites (see Regulatory Built /Ensembl; Additional file 1: Table S1) might affect transcription and translation; this still needs to be assessed.

The variant allele at rs4686302 (T) was previously shown to be associated with prematurity [8]. Our data showing an increased stimulatory effect of OXT in the samples with this variant allele goes along with this clinical finding as an increased stimulatory effect of OXT during preterm labor might lead to preterm delivery. Another indication that OXT sensitivity might be influenced by this sequence variant derives from an American study in which several allele variants were characterized in women requiring extreme OXT doses [18]. rs4686302 is a common substitution variant in exon 3 with nonsynonymous influence on the OXTR protein. The protein corresponding to this variant has a p.A218T amino acid substitution located in the transmembrane domain 5 that seems to be involved in the important step of G-protein activation together with the second intracellular loop and the third intracellular loop [19]. Therefore, a small change in the tertiary structure at this place could have consequences for G-protein-coupled signal transduction. An in silico prediction, however, revealed no functional significance of this amino acid substitution [8]. Since this variant was furthermore not associated with differences in the amounts of *OXTR*-mRNA in the current study, the meaning of the variant rs4686302 for OXT-induced cell signaling remains unknown. Also, in the case of this variant, no eQTL data were available (www.gtexportal.org/home, 28.09.2018); nevertheless, a CpG Island is located at the rs4686302 sequence, which might still influence expression regulation (Additional file 1: Table S1).

Labor is a complex process and numerous factors play important roles. The existence of genetically variant forms of OXTR are likely to influence patient responses to medications that bind to this receptor. Concerning treatment with synthetic OXT, clinical practice shows that not all patients benefit equally. Patients’ response to this medication is highly variable; some patients need higher, others lower doses to obtain a similar effect. It is conceivable that a screening method could enable personalized improvements in labor management. Also, the prevention of preterm delivery, which has a major impact on neonatal morbidity and mortality, might in the future profit from a personalized risk assessment of every patient. It is questionable if oxytocin-receptorantagonists for the treatment of preterm labor work equally well in every patient; clinical experience and first in vitro data such as ours suggest that subgroups of patients with specific single-nucleotide sequence variants of the *OXTR* benefit differently from diverse tocolytic agents. Our work, performed with myometrium samples from 60 patients, suggests that patients with rs237888 and/or rs4686302 may be more sensitive to OXT stimulation. This might explain why patients with these variants less often need a cesarean in the course of labor induction with OXT [11] and were thought to be prone to preterm labor [8], respectively]. Additional research should estimate possible clinical benefits of introducing an rs237888 and rs4686302 screening at pregnancy begin. While doing so and given that these two sequence variants are not in LD, it would make sense to consider them separately.

## Conclusion

Taken together, our data confirm that genetic variants of the *OXTR* gene might have an impact on the birth process and suggest that in some cases this occurs via differences in myometrial contractility strength as induced by OXT. Our work depicts the necessity of starting a discussion on whether introducing an *OXTR* variant screening at pregnancy begin would contribute to reduce the prevalence of preterm deliveries and/or of cesareans in the course of labor induction with OXT. A screening feasibility study could contribute to address this question. Moreover, additional basic research is needed to clarify how exactly the various *OXTR*-variants may influence contractility.

## Supplementary information

**Additional file 1: Table S1.** Additional annotations and functional data extracted from SNPnexus on the allele distribution of eight common *OXTR* variants previously shown to be associated with a relevant perinatal outcome.

**Additional file 2: Table S2.** Link disequilibrium values of the eight common *OXTR* sequence variants previously shown to be associated with a relevant perinatal outcome as determined using LDlink 3.0.

**Additional file 3: Table S3.** Distribution of the various sequence variant alleles in the study population.

**Additional file 4: Table S4. **OXTR-mRNA in the various reference and variant allele groups.

## Data Availability

The datasets used and/or analyzed during the current study are available from the corresponding author on reasonable request.
