# Supplementary material for: Influence of oxytocin receptor single nucleotide sequence variants on contractility of human myometrium: an in vitro functional study
Source: BMC Med Genet. 2019 Nov 12;20:178. doi: 10.1186/s12881-019-0894-8 (PMC6852767; doi:10.1186/s12881-019-0894-8)
Supplement: Supplementary file 1 — Additional file 1: Table S1. Additional annotations and functional data extracted from SNPnexus on the allele distribution of eight common OXTR variants previously shown to be associated with a relevant perinatal outcome. [file 12881_2019_894_MOESM1_ESM.pdf]

ID	SNP	Chromosome	Position	REF Allele	ALT Allele	Contig	Contig Position	Band	dbSNP
chr3:8752859:G/C/T:1	rs1042778	chr3	8752859	G	Y	GL000033.2	8742859	p25.3	rs1042778
chr3:8754861:A/C:1	rs11706648	chr3	8754861	A	C	GL000033.2	8744861	p25.3	rs11706648
chr3:8755409:T/C:1	rs237888	chr3	8755409	T	C	GL000033.2	8745409	p25.3	rs237888
chr3:8756900:C/T:1	rs4686301	chr3	8756900	C	T	GL000033.2	8746900	p25.3	rs4686301
chr3:8762685:A/G:1	rs53576	chr3	8762685	A	G	GL000033.2	8752685	p25.3	rs53576
chr3:8765737:T/C:1	rs237895	chr3	8765737	T	C	GL000033.2	8755737	p25.3	rs237895
chr3:8767498:G/A:1	rs237902	chr3	8767498	G	A	GL000033.2	8757498	p25.3	rs237902
chr3:8767536:C/T:1	rs4686302	chr3	8767536	C	T	GL000033.2	8757536	p25.3	rs4686302

ID	SNP	Chromosome	Position	Overlapped Gene	Type	Annotation
chr3:8752859:G/C/T:1	rs1042778	chr3	8752859	CAV3	protein_coding	non-coding intronic
chr3:8752859:G/C/T:1	rs1042778	chr3	8752859	OXTR	protein_coding	3utr
chr3:8754861:A/C:1	rs11706648	chr3	8754861	CAV3	protein_coding	non-coding intronic
chr3:8754861:A/C:1	rs11706648	chr3	8754861	OXTR	protein_coding	intronic
chr3:8755409:T/C:1	rs237888	chr3	8755409	CAV3	protein_coding	non-coding intronic
chr3:8755409:T/C:1	rs237888	chr3	8755409	OXTR	protein_coding	intronic
chr3:8756900:C/T:1	rs4686301	chr3	8756900	CAV3	protein_coding	non-coding intronic
chr3:8756900:C/T:1	rs4686301	chr3	8756900	OXTR	protein_coding	intronic
chr3:8762685:A/G:1	rs53576	chr3	8762685	CAV3	protein_coding	non-coding intronic
chr3:8762685:A/G:1	rs53576	chr3	8762685	OXTR	protein_coding	intronic
chr3:8765737:T/C:1	rs237895	chr3	8765737	CAV3	protein_coding	non-coding intronic
chr3:8765737:T/C:1	rs237895	chr3	8765737	OXTR	protein_coding	intronic
chr3:8767498:G/A:1	rs237902	chr3	8767498	CAV3	protein_coding	non-coding intronic
chr3:8767498:G/A:1	rs237902	chr3	8767498	OXTR	protein_coding	coding syn,3downstream
chr3:8767536:C/T:1	rs4686302	chr3	8767536	CAV3	protein_coding	non-coding intronic
chr3:8767536:C/T:1	rs4686302	chr3	8767536	OXTR	protein_coding	coding nonsyn,3downstream

SNP	Variant	Strand	Gene	Transcript	Entrez gene	Predicted Function	CDNA Position	CDS Position	AA Position	AA Change	Detail	Splice Distance
rs1042778	G|Y	1	OXTR	NM_000916	5021	3utr	1910					
rs1042778	G|Y	1	OXTR	NM_001354653	5021	3utr	1830					
rs1042778	G|Y	1	OXTR	NM_001354654	5021	3utr	1887					
rs1042778	G|Y	1	OXTR	NM_001354655	5021	3utr	1711					
rs1042778	G|Y	1	OXTR	NM_001354656	5021	3utr	1565					
rs11706648	A|C	1	OXTR	NM_000916	5021	intronic						1637
rs11706648	A|C	1	OXTR	NM_001354653	5021	intronic						1637
rs11706648	A|C	1	OXTR	NM_001354654	5021	intronic						1637
rs11706648	A|C	1	OXTR	NM_001354655	5021	intronic						1637
rs11706648	A|C	1	OXTR	NM_001354656	5021	intronic						1637
rs237888	T|C	1	OXTR	NM_000916	5021	intronic						2185
rs237888	T|C	1	OXTR	NM_001354653	5021	intronic						2185
rs237888	T|C	1	OXTR	NM_001354654	5021	intronic						2185
rs237888	T|C	1	OXTR	NM_001354655	5021	intronic						2185
rs237888	T|C	1	OXTR	NM_001354656	5021	intronic						2185
rs237895	T|C	1	OXTR	NM_000916	5021	intronic						1529
rs237895	T|C	1	OXTR	NM_001354653	5021	intronic						1529
rs237895	T|C	1	OXTR	NM_001354654	5021	intronic						1529
rs237895	T|C	1	OXTR	NM_001354655	5021	intronic						1529
rs237895	T|C	1	OXTR	NM_001354656	5021	intronic						1529
rs237902	G|A	1	OXTR	NM_000916	5021	coding	1312	690	230	N>N	syn	
rs237902	G|A	1	OXTR	NM_001354653	5021	coding	1232	690	230	N>N	syn	
rs237902	G|A	1	OXTR	NM_001354654	5021	coding	1289	690	230	N>N	syn	
rs237902	G|A	1	OXTR	NM_001354655	5021	coding	1113	690	230	N>N	syn	
rs237902	G|A	1	OXTR	NM_001354656	5021	coding	967	690	230	N>N	syn	
rs4686301	C|T	1	OXTR	NM_000916	5021	intronic						3676
rs4686301	C|T	1	OXTR	NM_001354653	5021	intronic						3676
rs4686301	C|T	1	OXTR	NM_001354654	5021	intronic						3676
rs4686301	C|T	1	OXTR	NM_001354655	5021	intronic						3676
rs4686301	C|T	1	OXTR	NM_001354656	5021	intronic						3676
rs4686302	C|T	1	OXTR	NM_000916	5021	coding	1274	652	218	A>T	nonsyn	
rs4686302	C|T	1	OXTR	NM_001354653	5021	coding	1194	652	218	A>T	nonsyn	
rs4686302	C|T	1	OXTR	NM_001354654	5021	coding	1251	652	218	A>T	nonsyn	
rs4686302	C|T	1	OXTR	NM_001354655	5021	coding	1075	652	218	A>T	nonsyn	
rs4686302	C|T	1	OXTR	NM_001354656	5021	coding	929	652	218	A>T	nonsyn	
rs53576	A|G	1	OXTR	NM_000916	5021	intronic						4581
rs53576	A|G	1	OXTR	NM_001354653	5021	intronic						4581
rs53576	A|G	1	OXTR	NM_001354654	5021	intronic						4581
rs53576	A|G	1	OXTR	NM_001354655	5021	intronic						4581
rs53576	A|G	1	OXTR	NM_001354656	5021	intronic						4581

SNP	Variant	Strand	Symbol	Gene	Predicted Function	CDNA Position	CDS Position	AA Position	AA Change	Detail	Splice Distance
rs1042778	G|Y	1	OXTR	uc003brc.4	3utr	1913					
rs1042778	G|Y	1	CAV3	uc062ghm.1	non-coding intronic						18869
rs11706648	A|C	1	OXTR	uc003brc.4	intronic						1637
rs11706648	A|C	1	CAV3	uc062ghm.1	non-coding intronic						20871
rs237888	T|C	1	OXTR	uc003brc.4	intronic						2185
rs237888	T|C	1	CAV3	uc062ghm.1	non-coding intronic						21419
rs237895	T|C	1	OXTR	uc003brc.4	intronic						1529
rs237895	T|C	1	CAV3	uc062ghm.1	non-coding intronic						11740
rs237902	G|A	1	OXTR	uc003brc.4	coding	1315	690	230	N>N	syn	
rs237902	G|A	1	CAV3	uc062ghm.1	non-coding intronic						9979
rs237902	G|A	1	OXTR	uc062ghn.1	3downstream						
rs237902	G|A	1	OXTR	uc062gho.1	3downstream						
rs237902	G|A	1	OXTR	uc062ghp.1	3downstream						
rs4686301	C|T	1	OXTR	uc003brc.4	intronic						3676
rs4686301	C|T	1	CAV3	uc062ghm.1	non-coding intronic						20577
rs4686302	C|T	1	OXTR	uc003brc.4	coding	1277	652	218	A>T	nonsyn	
rs4686302	C|T	1	CAV3	uc062ghm.1	non-coding intronic						9941
rs4686302	C|T	1	OXTR	uc062ghn.1	3downstream						
rs4686302	C|T	1	OXTR	uc062gho.1	3downstream						
rs4686302	C|T	1	OXTR	uc062ghp.1	3downstream						
rs53576	A|G	1	OXTR	uc003brc.4	intronic						4581
rs53576	A|G	1	CAV3	uc062ghm.1	non-coding intronic						14792

SNP	Variant	Strand	Symbol	Gene	Transcript	Predicted Function	CDNA Position	CDS Position	AA Position	AA Change	Detail	Splice Distance
rs1042778	G|Y	1	OXTR	ENSG00000180914	ENST00000316793	3utr	1913					
rs1042778	G|Y	1	CAV3	ENSG00000182533	ENST00000472766	non-coding intronic						18869
rs11706648	A|C	1	OXTR	ENSG00000180914	ENST00000316793	intronic						1637
rs11706648	A|C	1	CAV3	ENSG00000182533	ENST00000472766	non-coding intronic						20871
rs237888	T|C	1	OXTR	ENSG00000180914	ENST00000316793	intronic						2185
rs237888	T|C	1	CAV3	ENSG00000182533	ENST00000472766	non-coding intronic						21419
rs237895	T|C	1	OXTR	ENSG00000180914	ENST00000316793	intronic						1529
rs237895	T|C	1	CAV3	ENSG00000182533	ENST00000472766	non-coding intronic						11740
rs237902	G|A	1	OXTR	ENSG00000180914	ENST00000316793	coding	1315	690	230	N>N	syn	
rs237902	G|A	1	OXTR	ENSG00000180914	ENST00000431493	3downstream						
rs237902	G|A	1	OXTR	ENSG00000180914	ENST00000449615	3downstream						
rs237902	G|A	1	CAV3	ENSG00000182533	ENST00000472766	non-coding intronic						9979
rs237902	G|A	1	OXTR	ENSG00000180914	ENST00000474615	3downstream						
rs4686301	C|T	1	OXTR	ENSG00000180914	ENST00000316793	intronic						3676
rs4686301	C|T	1	CAV3	ENSG00000182533	ENST00000472766	non-coding intronic						20577
rs4686302	C|T	1	OXTR	ENSG00000180914	ENST00000316793	coding	1277	652	218	A>T	nonsyn	
rs4686302	C|T	1	OXTR	ENSG00000180914	ENST00000431493	3downstream						
rs4686302	C|T	1	OXTR	ENSG00000180914	ENST00000449615	3downstream						
rs4686302	C|T	1	CAV3	ENSG00000182533	ENST00000472766	non-coding intronic						9941
rs4686302	C|T	1	OXTR	ENSG00000180914	ENST00000474615	3downstream						
rs53576	A|G	1	OXTR	ENSG00000180914	ENST00000316793	intronic						4581
rs53576	A|G	1	CAV3	ENSG00000182533	ENST00000472766	non-coding intronic						14792

SNP	Variant	Transcript	Protein	AA Position	wild_AA	mutant_AA	Score	Prediction
rs4686302	C|T	ENST00000316793	ENSP00000324270	218	A	T	0.4	tolerated

SNP	Variant	Transcript	Protein	AA Position	wild_AA	mutant_AA	Score	Prediction
rs4686302	C|T	ENST00000316793	ENSP00000324270	218	A	T	0.01	benign

SNP	Chrom	Position	REF Allele	ALT Allele	Frequency
rs1042778	3	8752859	G	T	0.3767
rs11706648	3	8754861	A	C	0.3201
rs237888	3	8755409	T	C	0.0596
rs4686301	3	8756900	C	T	0.3082
rs53576	3	8762685	A	G	0.6491
rs237895	3	8765737	T	C	0.5895
rs237902	3	8767498	G	A	0.327
rs4686302	3	8767536	C	T	0.1183

SNP	Chromosome	chromStart	chromEnd	CpG_Island	Length	CpG %	C/G %	Ratio
rs237902	chr3	8767275	8769594	CpG: 184	2319	15.9	63.6	0.79
rs4686302	chr3	8767275	8769594	CpG: 184	2319	15.9	63.6	0.79

SNP	Chromosome	Region Start	Region End	Feature Type Class	Feature Type	Epigenome
rs1042778	chr3	8750440	8753120	Histone	H3K36me3	HSMM
rs1042778	chr3	8751420	8757980	Histone	H3K79me2	HSMM
rs1042778	chr3	8752000	8754800	Histone	H3K36me3	HSMMtube
rs1042778	chr3	8752140	8753300	Histone	H3K36me3	NH-A
rs1042778	chr3	8752200	8753760	Histone	H3K36me3	DND-41
rs1042778	chr3	8752320	8753840	Histone	H3K79me2	HSMMtube
rs1042778	chr3	8752360	8754180	Histone	H3K36me3	GM12878
rs1042778	chr3	8752700	8753300	Histone	H4K20me1	NH-A
rs1042778	chr3	8752780	8753180	Histone	H3K36me3	A549
rs11706648	chr3	8751420	8757980	Histone	H3K79me2	HSMM
rs11706648	chr3	8753200	8754900	Histone	H3K36me3	HSMM
rs11706648	chr3	8754300	8754880	Histone	H3K4me1	HSMMtube
rs11706648	chr3	8754520	8754880	Histone	H4K20me1	NH-A
rs11706648	chr3	8754560	8756540	Histone	H3K4me1	HepG2
rs237888	chr3	8751420	8757980	Histone	H3K79me2	HSMM
rs237888	chr3	8754560	8756540	Histone	H3K4me1	HepG2
rs237888	chr3	8754920	8757260	Histone	H3K36me3	HSMM
rs237888	chr3	8755029	8755472	Transcription Factor	FOXA1	HepG2
rs237888	chr3	8755080	8755500	Histone	H3K79me2	HSMMtube
rs237888	chr3	8755200	8755560	Histone	H3K36me3	Osteobl
rs237888	chr3	8755220	8755440	Histone	H3K4me1	A549
rs237888	chr3	8755240	8755780	Histone	H4K20me1	Osteobl
rs237888	chr3	8755359	8756021	Transcription Factor	CTCF	HSMM
rs237888	chr3	8755389	8755988	Transcription Factor	CTCF	Osteobl
rs4686301	chr3	8751420	8757980	Histone	H3K79me2	HSMM
rs4686301	chr3	8754920	8757260	Histone	H3K36me3	HSMM
rs4686301	chr3	8755860	8757240	Histone	H3K79me2	HSMMtube
rs4686301	chr3	8755940	8757260	Histone	H3K4me1	HSMMtube
rs4686301	chr3	8756420	8758280	Histone	H3K4me1	HSMM
rs4686301	chr3	8756460	8757260	Histone	H3K4me1	NHDF-AD
rs4686301	chr3	8756520	8757000	Histone	H3K4me1	NH-A
rs4686301	chr3	8756569	8757033	Open Chromatin	DNase1	HSMMtube
rs4686301	chr3	8756577	8757043	Open Chromatin	DNase1	NHDF-AD
rs4686301	chr3	8756840	8757140	Histone	H3K4me1	HMEC
rs53576	chr3	8759780	8769580	Histone	H3K79me2	HSMM
rs53576	chr3	8762360	8762800	Histone	H3K36me3	HSMM
rs53576	chr3	8762400	8763640	Histone	H3K4me1	Osteobl
rs53576	chr3	8762400	8763880	Histone	H3K79me2	HSMMtube
rs53576	chr3	8762460	8762860	Histone	H4K20me1	Osteobl
rs53576	chr3	8762460	8762740	Histone	H3K27me3	HepG2
rs53576	chr3	8762480	8763380	Histone	H3K4me1	HepG2
rs53576	chr3	8762540	8764980	Histone	H3K4me1	HSMM
rs53576	chr3	8762540	8763420	Histone	H4K20me1	NH-A
rs53576	chr3	8762620	8763760	Histone	H3K4me1	HSMMtube
rs237895	chr3	8759780	8769580	Histone	H3K79me2	HSMM
rs237895	chr3	8763840	8766100	Histone	H3K36me3	HSMM
rs237895	chr3	8763940	8769500	Histone	H3K4me1	NH-A
rs237895	chr3	8763960	8770080	Histone	H3K79me2	HSMMtube
rs237895	chr3	8764760	8766820	Histone	H3K4me1	HepG2
rs237895	chr3	8764903	8770258	Histone	H3K4me2	NH-A
rs237895	chr3	8765000	8768000	Histone	H3K4me1	HSMM
rs237895	chr3	8765000	8769500	Histone	H3K79me2	GM12878
rs237895	chr3	8765040	8771940	Histone	H3K27me3	Monocytes-CD14+
rs237895	chr3	8765180	8766160	Histone	H4K20me1	NH-A
rs237895	chr3	8765239	8769523	Histone	H3K4me2	HSMM
rs237895	chr3	8765300	8766160	Histone	H3K27me3	HUVEC
rs237895	chr3	8765440	8765820	Histone	H3K4me1	Osteobl
rs237895	chr3	8765540	8765800	Histone	H3K36me3	GM12878
rs237895	chr3	8765540	8765780	Histone	H3K27me3	HepG2
rs237895	chr3	8765625	8770033	Histone	H3K4me3	HSMM

rs237895	chr3	8765660	8766040	Histone	H4K20me1	Osteobl
rs237902	chr3	8759780	8769580	Histone	H3K79me2	HSMM
rs237902	chr3	8763940	8769500	Histone	H3K4me1	NH-A
rs237902	chr3	8763960	8770080	Histone	H3K79me2	HSMMtube
rs237902	chr3	8764903	8770258	Histone	H3K4me2	NH-A
rs237902	chr3	8765000	8768000	Histone	H3K4me1	HSMM
rs237902	chr3	8765000	8769500	Histone	H3K79me2	GM12878
rs237902	chr3	8765040	8771940	Histone	H3K27me3	Monocytes-CD14+
rs237902	chr3	8765239	8769523	Histone	H3K4me2	HSMM
rs237902	chr3	8765625	8770033	Histone	H3K4me3	HSMM
rs237902	chr3	8765980	8768140	Histone	H3K4me1	NHLF
rs237902	chr3	8765983	8770171	Histone	H3K4me3	Osteobl
rs237902	chr3	8766100	8769740	Histone	H3K27me3	NHEK
rs237902	chr3	8766180	8769500	Histone	H3K27me3	HUVEC
rs237902	chr3	8766200	8767680	Histone	H4K20me1	NH-A
rs237902	chr3	8766240	8768120	Histone	H3K36me3	NHLF
rs237902	chr3	8766264	8769562	Histone	H3K4me3	NH-A
rs237902	chr3	8766273	8769530	Histone	H3K9ac	NH-A
rs237902	chr3	8766306	8769956	Histone	H3K4me3	GM12878
rs237902	chr3	8766340	8767540	Histone	H3K36me3	HSMM
rs237902	chr3	8766580	8768120	Histone	H3K27me3	Osteobl
rs237902	chr3	8766759	8770010	Histone	H3K4me2	GM12878
rs237902	chr3	8766780	8770040	Histone	H3K4me1	GM12878
rs237902	chr3	8767140	8768140	Histone	H3K4me1	NHDF-AD
rs237902	chr3	8767214	8769840	Open Chromatin	DNase1	H1ESC
rs237902	chr3	8767220	8767540	Histone	H3K4me1	K562
rs237902	chr3	8767226	8770004	Histone	H3K4me2	NHDF-AD
rs237902	chr3	8767260	8770120	Histone	H3K4me1	HMEC
rs237902	chr3	8767275	8769966	Histone	H2AZ	GM12878
rs237902	chr3	8767292	8768330	Transcription Factor	ZEB1	GM12878
rs237902	chr3	8767340	8768300	Histone	H3K36me3	DND-41
rs237902	chr3	8767380	8768180	Histone	H3K4me1	Osteobl
rs237902	chr3	8767414	8768687	Histone	H3K4me2	HMEC
rs237902	chr3	8767449	8769855	Histone	H3K4me2	NHEK
rs237902	chr3	8767462	8770076	Histone	H3K4me2	H1ESC
rs237902	chr3	8767492	8768280	Transcription Factor	CTCF	NHEK

SNP	Chromosome	Region Start	Region End	Feature Type Class	Feature Type	Epigenome
rs1042778	chr3	8752320	8753140	Histone	H3K36me3	Fetal Muscle Leg
rs1042778	chr3	8752320	8753140	Histone	H3K36me3	Fetal Muscle Leg
rs1042778	chr3	8752320	8754200	Histone	H3K36me3	H1-mesenchymal
rs1042778	chr3	8752320	8754200	Histone	H3K36me3	H1-mesenchymal
rs1042778	chr3	8752340	8755000	Histone	H3K4me1	Fetal Muscle Leg
rs1042778	chr3	8752340	8755000	Histone	H3K4me1	Fetal Muscle Leg
rs1042778	chr3	8752500	8753380	Histone	H3K36me3	Fetal Stomach
rs1042778	chr3	8752500	8753380	Histone	H3K36me3	Fetal Stomach
rs1042778	chr3	8752680	8753560	Histone	H3K36me3	H1-trophoblast
rs1042778	chr3	8752680	8753560	Histone	H3K36me3	H1-trophoblast
rs1042778	chr3	8752720	8754160	Histone	H3K27me3	Fetal Adrenal Gland
rs1042778	chr3	8752720	8754160	Histone	H3K27me3	Fetal Adrenal Gland
rs1042778	chr3	8752720	8752980	Histone	H3K9me3	Lung
rs1042778	chr3	8752720	8752980	Histone	H3K9me3	Lung
rs1042778	chr3	8752760	8753200	Histone	H3K36me3	Lung
rs1042778	chr3	8752760	8753200	Histone	H3K36me3	Lung
rs1042778	chr3	8752800	8753680	Histone	H3K4me1	iPS DF 19.11
rs1042778	chr3	8752800	8753680	Histone	H3K4me1	iPS DF 19.11
rs1042778	chr3	8752800	8753640	Histone	H3K4me1	H1-mesenchymal
rs1042778	chr3	8752800	8753640	Histone	H3K4me1	H1-mesenchymal
rs11706648	chr3	8752340	8755000	Histone	H3K4me1	Fetal Muscle Leg
rs11706648	chr3	8752340	8755000	Histone	H3K4me1	Fetal Muscle Leg
rs237888	chr3	8755160	8755440	Histone	H3K27me3	iPS-20b
rs237888	chr3	8755380	8762640	Histone	H3K4me1	Fetal Muscle Leg
rs237888	chr3	8755380	8762640	Histone	H3K4me1	Fetal Muscle Leg
rs4686301	chr3	8755380	8762640	Histone	H3K4me1	Fetal Muscle Leg
rs4686301	chr3	8755380	8762640	Histone	H3K4me1	Fetal Muscle Leg
rs4686301	chr3	8756300	8757220	Histone	H3K27me3	Fetal Adrenal Gland
rs4686301	chr3	8756300	8757220	Histone	H3K27me3	Fetal Adrenal Gland
rs4686301	chr3	8756380	8761680	Histone	H3K4me1	Fetal Muscle Trunk
rs4686301	chr3	8756380	8761680	Histone	H3K4me1	Fetal Muscle Trunk
rs4686301	chr3	8756440	8757220	Histone	H3K36me3	H1-mesenchymal
rs4686301	chr3	8756440	8757220	Histone	H3K36me3	H1-mesenchymal
rs4686301	chr3	8756460	8757180	Histone	H3K4me1	H1-mesenchymal
rs4686301	chr3	8756460	8757180	Histone	H3K4me1	H1-mesenchymal
rs4686301	chr3	8756540	8757180	Histone	H3K4me1	iPS DF 19.11
rs4686301	chr3	8756540	8757180	Histone	H3K4me1	iPS DF 19.11
rs4686301	chr3	8756557	8759183	Histone	H3K27ac	Fetal Muscle Trunk
rs4686301	chr3	8756557	8759183	Histone	H3K27ac	Fetal Muscle Trunk
rs53576	chr3	8759460	8763860	Histone	H3K27me3	Fetal Adrenal Gland
rs53576	chr3	8759460	8763860	Histone	H3K27me3	Fetal Adrenal Gland
rs53576	chr3	8759560	8763840	Histone	H3K36me3	H1-mesenchymal
rs53576	chr3	8759560	8763840	Histone	H3K36me3	H1-mesenchymal
rs53576	chr3	8762380	8771680	Histone	H3K27me3	Fetal Stomach
rs53576	chr3	8762380	8771680	Histone	H3K27me3	Fetal Stomach
rs53576	chr3	8762440	8763760	Histone	H3K4me1	H1-mesenchymal
rs53576	chr3	8762440	8763760	Histone	H3K4me1	H1-mesenchymal
rs53576	chr3	8762480	8763880	Histone	H3K27me3	Placenta
rs53576	chr3	8762480	8763880	Histone	H3K27me3	Placenta
rs53576	chr3	8762620	8763440	Histone	H3K4me1	iPS DF 19.11
rs53576	chr3	8762620	8763440	Histone	H3K4me1	iPS DF 19.11
rs237895	chr3	8762380	8771680	Histone	H3K27me3	Fetal Stomach
rs237895	chr3	8762380	8771680	Histone	H3K27me3	Fetal Stomach
rs237895	chr3	8763720	8771160	Histone	H3K27me3	Fetal Thymus
rs237895	chr3	8763720	8771160	Histone	H3K27me3	Fetal Thymus
rs237895	chr3	8763900	8770900	Histone	H3K27me3	iPS-20b
rs237895	chr3	8763980	8766120	Histone	H3K36me3	H1-mesenchymal
rs237895	chr3	8763980	8766120	Histone	H3K36me3	H1-mesenchymal
rs237895	chr3	8764020	8766900	Histone	H3K4me1	Fetal Muscle Trunk
rs237895	chr3	8764020	8766900	Histone	H3K4me1	Fetal Muscle Trunk
rs237895	chr3	8764820	8770060	Histone	H3K4me1	Fetal Muscle Leg
rs237895	chr3	8764820	8770060	Histone	H3K4me1	Fetal Muscle Leg
rs237895	chr3	8764980	8765940	Histone	H3K27me3	Monocytes-CD14+ (PB) Roadmap
rs237895	chr3	8764980	8765940	Histone	H3K27me3	Monocytes-CD14+ (PB) Roadmap
rs237895	chr3	8765380	8766060	Histone	H3K27me3	Fetal Intestine Large
rs237895	chr3	8765380	8766060	Histone	H3K27me3	Fetal Intestine Large
rs237895	chr3	8765500	8765980	Histone	H3K4me1	H1-mesenchymal
rs237895	chr3	8765500	8765980	Histone	H3K4me1	H1-mesenchymal

rs237895	chr3	8765580	8766900	Histone	H3K27me3	Placenta
rs237895	chr3	8765580	8766900	Histone	H3K27me3	Placenta
rs237895	chr3	8765600	8771200	Histone	H3K27me3	Fetal Muscle Trunk
rs237895	chr3	8765600	8771200	Histone	H3K27me3	Fetal Muscle Trunk
rs237902	chr3	8762380	8771680	Histone	H3K27me3	Fetal Stomach
rs237902	chr3	8762380	8771680	Histone	H3K27me3	Fetal Stomach
rs237902	chr3	8763720	8771160	Histone	H3K27me3	Fetal Thymus
rs237902	chr3	8763720	8771160	Histone	H3K27me3	Fetal Thymus
rs237902	chr3	8763900	8770900	Histone	H3K27me3	iPS-20b
rs237902	chr3	8764820	8770060	Histone	H3K4me1	Fetal Muscle Leg
rs237902	chr3	8764820	8770060	Histone	H3K4me1	Fetal Muscle Leg
rs237902	chr3	8765600	8771200	Histone	H3K27me3	Fetal Muscle Trunk
rs237902	chr3	8765600	8771200	Histone	H3K27me3	Fetal Muscle Trunk
rs237902	chr3	8765980	8771600	Histone	H3K27me3	Monocytes-CD14+ (PB) Roadmap
rs237902	chr3	8765980	8771600	Histone	H3K27me3	Monocytes-CD14+ (PB) Roadmap
rs237902	chr3	8766020	8768600	Histone	H3K4me1	H1-mesenchymal
rs237902	chr3	8766020	8768600	Histone	H3K4me1	H1-mesenchymal
rs237902	chr3	8766040	8769820	Histone	H3K27me3	Fetal Adrenal Gland
rs237902	chr3	8766040	8769820	Histone	H3K27me3	Fetal Adrenal Gland
rs237902	chr3	8766080	8771020	Histone	H3K27me3	Fetal Intestine Large
rs237902	chr3	8766080	8771020	Histone	H3K27me3	Fetal Intestine Large
rs237902	chr3	8766100	8771120	Histone	H3K27me3	Fetal Muscle Leg
rs237902	chr3	8766100	8771120	Histone	H3K27me3	Fetal Muscle Leg
rs237902	chr3	8766140	8771100	Histone	H3K27me3	Fetal Intestine Small
rs237902	chr3	8766140	8771100	Histone	H3K27me3	Fetal Intestine Small
rs237902	chr3	8766160	8768340	Histone	H3K36me3	H1-mesenchymal
rs237902	chr3	8766160	8768340	Histone	H3K36me3	H1-mesenchymal
rs237902	chr3	8766240	8769460	Histone	H2AK5ac	IMR90
rs237902	chr3	8766500	8769820	Histone	H3K27me3	H1ESC
rs237902	chr3	8766500	8769540	Histone	H3K27me3	H1ESC
rs237902	chr3	8766880	8769700	Histone	H3K4me1	iPS DF 19.11
rs237902	chr3	8766880	8769700	Histone	H3K4me1	iPS DF 19.11
rs237902	chr3	8766980	8767600	Histone	H3K27me3	Placenta
rs237902	chr3	8766980	8767600	Histone	H3K27me3	Placenta
rs237902	chr3	8767020	8769780	Histone	H3K4me1	Fetal Muscle Trunk
rs237902	chr3	8767020	8769780	Histone	H3K4me1	Fetal Muscle Trunk
rs237902	chr3	8767020	8771100	Histone	H3K27me3	T cells (PB) Roadmap
rs237902	chr3	8767020	8771100	Histone	H3K27me3	T cells (PB) Roadmap
rs237902	chr3	8767100	8768680	Histone	H3K4me1	IMR90
rs237902	chr3	8767100	8769780	Histone	H3K4me1	Lung
rs237902	chr3	8767100	8769780	Histone	H3K4me1	Lung
rs237902	chr3	8767160	8768580	Histone	H3K4me1	Pancreas
rs237902	chr3	8767160	8768580	Histone	H3K4me1	Pancreas
rs237902	chr3	8767200	8768360	Histone	H3K4me1	Gastric
rs237902	chr3	8767200	8768360	Histone	H3K4me1	Gastric
rs237902	chr3	8767220	8767980	Histone	H3K36me3	H1-trophoblast
rs237902	chr3	8767220	8767980	Histone	H3K36me3	H1-trophoblast
rs237902	chr3	8767240	8767920	Histone	H3K9me3	iPS DF 19.11
rs237902	chr3	8767240	8767920	Histone	H3K9me3	iPS DF 19.11
rs237902	chr3	8767260	8769520	Histone	H3K4me1	H1-trophoblast
rs237902	chr3	8767260	8769520	Histone	H3K4me1	H1-trophoblast
rs237902	chr3	8767280	8769820	Histone	H3K4me1	Spleen
rs237902	chr3	8767280	8769820	Histone	H3K4me1	Spleen
rs237902	chr3	8767300	8767980	Histone	H3K9me3	Lung
rs237902	chr3	8767300	8767980	Histone	H3K9me3	Lung
rs237902	chr3	8767340	8767980	Histone	H3K4me1	Psoas Muscle
rs237902	chr3	8767380	8768020	Histone	H3K27me3	H1-trophoblast
rs237902	chr3	8767380	8768020	Histone	H3K27me3	H1-trophoblast
rs237902	chr3	8767400	8767920	Histone	H3K4me1	iPS DF 6.9
rs237902	chr3	8767400	8767920	Histone	H3K4me1	iPS DF 6.9
rs237902	chr3	8767400	8767560	Histone	H3K27me3	H1-neuronal progenitor
rs237902	chr3	8767400	8767560	Histone	H3K27me3	H1-neuronal progenitor
rs237902	chr3	8767420	8767920	Histone	H3K4me1	Left Ventricle
rs237902	chr3	8767420	8767920	Histone	H3K4me1	Left Ventricle
rs237902	chr3	8767436	8769990	Histone	H3K4me2	IMR90
rs237902	chr3	8767440	8767720	Histone	H3K14ac	IMR90
rs237902	chr3	8767473	8769957	Histone	H3K4me3	IMR90

SNP	Chromosome	Region Start	Region End	Feature Type Class	Epigenome	Activity
rs237888	chr3	8755401	8756000	CTCF Binding Site	A549	ACTIVE
rs237888	chr3	8755401	8756000	CTCF Binding Site	Aorta	INACTIVE
rs237888	chr3	8755401	8756000	CTCF Binding Site	Aorta	INACTIVE
rs237888	chr3	8755401	8756000	CTCF Binding Site	B cells (PB) Roadmap	INACTIVE
rs237888	chr3	8755401	8756000	CTCF Binding Site	B cells (PB) Roadmap	INACTIVE
rs237888	chr3	8755401	8756000	CTCF Binding Site	CD14+CD16- monocyte (CB)	INACTIVE
rs237888	chr3	8755401	8756000	CTCF Binding Site	CD14+CD16- monocyte (VB)	INACTIVE
rs237888	chr3	8755401	8756000	CTCF Binding Site	CD4+ ab T cell (VB)	INACTIVE
rs237888	chr3	8755401	8756000	CTCF Binding Site	CD8+ ab T cell (CB)	INACTIVE
rs237888	chr3	8755401	8756000	CTCF Binding Site	CM CD4+ ab T cell (VB)	INACTIVE
rs237888	chr3	8755401	8756000	CTCF Binding Site	DND-41	ACTIVE
rs237888	chr3	8755401	8756000	CTCF Binding Site	eosinophil (VB)	INACTIVE
rs237888	chr3	8755401	8756000	CTCF Binding Site	EPC (VB)	INACTIVE
rs237888	chr3	8755401	8756000	CTCF Binding Site	erythroblast (CB)	INACTIVE
rs237888	chr3	8755401	8756000	CTCF Binding Site	Fetal Adrenal Gland	INACTIVE
rs237888	chr3	8755401	8756000	CTCF Binding Site	Fetal Adrenal Gland	INACTIVE
rs237888	chr3	8755401	8756000	CTCF Binding Site	Fetal Intestine Large	INACTIVE
rs237888	chr3	8755401	8756000	CTCF Binding Site	Fetal Intestine Large	INACTIVE
rs237888	chr3	8755401	8756000	CTCF Binding Site	Fetal Intestine Small	INACTIVE
rs237888	chr3	8755401	8756000	CTCF Binding Site	Fetal Intestine Small	INACTIVE
rs237888	chr3	8755401	8756000	CTCF Binding Site	Fetal Muscle Leg	INACTIVE
rs237888	chr3	8755401	8756000	CTCF Binding Site	Fetal Muscle Leg	INACTIVE
rs237888	chr3	8755401	8756000	CTCF Binding Site	Fetal Muscle Trunk	INACTIVE
rs237888	chr3	8755401	8756000	CTCF Binding Site	Fetal Muscle Trunk	INACTIVE
rs237888	chr3	8755401	8756000	CTCF Binding Site	Fetal Stomach	INACTIVE
rs237888	chr3	8755401	8756000	CTCF Binding Site	Fetal Stomach	INACTIVE
rs237888	chr3	8755401	8756000	CTCF Binding Site	Fetal Thymus	INACTIVE
rs237888	chr3	8755401	8756000	CTCF Binding Site	Fetal Thymus	INACTIVE
rs237888	chr3	8755401	8756000	CTCF Binding Site	Gastric	INACTIVE
rs237888	chr3	8755401	8756000	CTCF Binding Site	Gastric	INACTIVE
rs237888	chr3	8755401	8756000	CTCF Binding Site	GM12878	ACTIVE
rs237888	chr3	8755401	8756000	CTCF Binding Site	H1-mesenchymal	INACTIVE
rs237888	chr3	8755401	8756000	CTCF Binding Site	H1-mesenchymal	INACTIVE
rs237888	chr3	8755401	8756000	CTCF Binding Site	H1-neuronal progenitor	INACTIVE
rs237888	chr3	8755401	8756000	CTCF Binding Site	H1-neuronal progenitor	INACTIVE
rs237888	chr3	8755401	8756000	CTCF Binding Site	H1-trophoblast	INACTIVE
rs237888	chr3	8755401	8756000	CTCF Binding Site	H1-trophoblast	INACTIVE
rs237888	chr3	8755401	8756000	CTCF Binding Site	H1ESC	ACTIVE
rs237888	chr3	8755401	8756000	CTCF Binding Site	H9	INACTIVE
rs237888	chr3	8755401	8756000	CTCF Binding Site	H9	INACTIVE
rs237888	chr3	8755401	8756000	CTCF Binding Site	HeLa-S3	ACTIVE
rs237888	chr3	8755401	8756000	CTCF Binding Site	HepG2	INACTIVE
rs237888	chr3	8755401	8756000	CTCF Binding Site	HMEC	ACTIVE
rs237888	chr3	8755401	8756000	CTCF Binding Site	HSMM	INACTIVE
rs237888	chr3	8755401	8756000	CTCF Binding Site	HSMMtube	ACTIVE
rs237888	chr3	8755401	8756000	CTCF Binding Site	HUVEC	ACTIVE
rs237888	chr3	8755401	8756000	CTCF Binding Site	HUVEC prol (CB)	INACTIVE
rs237888	chr3	8755401	8756000	CTCF Binding Site	IMR90	ACTIVE
rs237888	chr3	8755401	8756000	CTCF Binding Site	iPS-20b	INACTIVE
rs237888	chr3	8755401	8756000	CTCF Binding Site	iPS DF 19.11	INACTIVE
rs237888	chr3	8755401	8756000	CTCF Binding Site	iPS DF 19.11	INACTIVE
rs237888	chr3	8755401	8756000	CTCF Binding Site	iPS DF 6.9	INACTIVE
rs237888	chr3	8755401	8756000	CTCF Binding Site	iPS DF 6.9	INACTIVE
rs237888	chr3	8755401	8756000	CTCF Binding Site	K562	ACTIVE
rs237888	chr3	8755401	8756000	CTCF Binding Site	Left Ventricle	INACTIVE
rs237888	chr3	8755401	8756000	CTCF Binding Site	Left Ventricle	INACTIVE
rs237888	chr3	8755401	8756000	CTCF Binding Site	Lung	INACTIVE
rs237888	chr3	8755401	8756000	CTCF Binding Site	Lung	INACTIVE
rs237888	chr3	8755401	8756000	CTCF Binding Site	M0 macrophage (CB)	INACTIVE
rs237888	chr3	8755401	8756000	CTCF Binding Site	M0 macrophage (VB)	INACTIVE
rs237888	chr3	8755401	8756000	CTCF Binding Site	M1 macrophage (CB)	INACTIVE
rs237888	chr3	8755401	8756000	CTCF Binding Site	M1 macrophage (VB)	INACTIVE
rs237888	chr3	8755401	8756000	CTCF Binding Site	M2 macrophage (CB)	INACTIVE
rs237888	chr3	8755401	8756000	CTCF Binding Site	M2 macrophage (VB)	INACTIVE
rs237888	chr3	8755401	8756000	CTCF Binding Site	Monocytes-CD14+	INACTIVE
rs237888	chr3	8755401	8756000	CTCF Binding Site	Monocytes-CD14+ (PB) Roadmap	INACTIVE
rs237888	chr3	8755401	8756000	CTCF Binding Site	Monocytes-CD14+ (PB) Roadmap	INACTIVE
rs237888	chr3	8755401	8756000	CTCF Binding Site	MSC (VB)	INACTIVE

rs237888	chr3	8755401	8756000	CTCF Binding Site	naive B cell (VB)	INACTIVE
rs237888	chr3	8755401	8756000	CTCF Binding Site	Natural Killer cells (PB)	INACTIVE
rs237888	chr3	8755401	8756000	CTCF Binding Site	Natural Killer cells (PB)	INACTIVE
rs237888	chr3	8755401	8756000	CTCF Binding Site	neutrophil (CB)	INACTIVE
rs237888	chr3	8755401	8756000	CTCF Binding Site	neutrophil myelocyte (BM)	INACTIVE
rs237888	chr3	8755401	8756000	CTCF Binding Site	neutrophil (VB)	INACTIVE
rs237888	chr3	8755401	8756000	CTCF Binding Site	NH-A	REPRESSED
rs237888	chr3	8755401	8756000	CTCF Binding Site	NHDF-AD	INACTIVE
rs237888	chr3	8755401	8756000	CTCF Binding Site	NHEK	ACTIVE
rs237888	chr3	8755401	8756000	CTCF Binding Site	NHLF	ACTIVE
rs237888	chr3	8755401	8756000	CTCF Binding Site	Osteobl	REPRESSED
rs237888	chr3	8755401	8756000	CTCF Binding Site	Ovary	INACTIVE
rs237888	chr3	8755401	8756000	CTCF Binding Site	Ovary	INACTIVE
rs237888	chr3	8755401	8756000	CTCF Binding Site	Pancreas	INACTIVE
rs237888	chr3	8755401	8756000	CTCF Binding Site	Pancreas	INACTIVE
rs237888	chr3	8755401	8756000	CTCF Binding Site	Placenta	INACTIVE
rs237888	chr3	8755401	8756000	CTCF Binding Site	Placenta	INACTIVE
rs237888	chr3	8755401	8756000	CTCF Binding Site	Psoas Muscle	INACTIVE
rs237888	chr3	8755401	8756000	CTCF Binding Site	Right Atrium	INACTIVE
rs237888	chr3	8755401	8756000	CTCF Binding Site	Right Atrium	INACTIVE
rs237888	chr3	8755401	8756000	CTCF Binding Site	Small Intestine	INACTIVE
rs237888	chr3	8755401	8756000	CTCF Binding Site	Small Intestine	INACTIVE
rs237888	chr3	8755401	8756000	CTCF Binding Site	Spleen	INACTIVE
rs237888	chr3	8755401	8756000	CTCF Binding Site	Spleen	INACTIVE
rs237888	chr3	8755401	8756000	CTCF Binding Site	T cells (PB) Roadmap	INACTIVE
rs237888	chr3	8755401	8756000	CTCF Binding Site	T cells (PB) Roadmap	INACTIVE
rs237888	chr3	8755401	8756000	CTCF Binding Site	Thymus	INACTIVE
rs237888	chr3	8755401	8756000	CTCF Binding Site	Thymus	INACTIVE
rs4686301	chr3	8756569	8757043	Open chromatin	A549	INACTIVE
rs4686301	chr3	8756569	8757043	Open chromatin	Aorta	NA
rs4686301	chr3	8756569	8757043	Open chromatin	Aorta	NA
rs4686301	chr3	8756569	8757043	Open chromatin	B cells (PB) Roadmap	NA
rs4686301	chr3	8756569	8757043	Open chromatin	B cells (PB) Roadmap	NA
rs4686301	chr3	8756569	8757043	Open chromatin	CD14+CD16- monocyte (CB)	NA
rs4686301	chr3	8756569	8757043	Open chromatin	CD14+CD16- monocyte (VB)	NA
rs4686301	chr3	8756569	8757043	Open chromatin	CD4+ ab T cell (VB)	NA
rs4686301	chr3	8756569	8757043	Open chromatin	CD8+ ab T cell (CB)	NA
rs4686301	chr3	8756569	8757043	Open chromatin	CM CD4+ ab T cell (VB)	NA
rs4686301	chr3	8756569	8757043	Open chromatin	DND-41	NA
rs4686301	chr3	8756569	8757043	Open chromatin	eosinophil (VB)	NA
rs4686301	chr3	8756569	8757043	Open chromatin	EPC (VB)	NA
rs4686301	chr3	8756569	8757043	Open chromatin	erythroblast (CB)	NA
rs4686301	chr3	8756569	8757043	Open chromatin	Fetal Adrenal Gland	NA
rs4686301	chr3	8756569	8757043	Open chromatin	Fetal Adrenal Gland	NA
rs4686301	chr3	8756569	8757043	Open chromatin	Fetal Intestine Large	NA
rs4686301	chr3	8756569	8757043	Open chromatin	Fetal Intestine Large	NA
rs4686301	chr3	8756569	8757043	Open chromatin	Fetal Intestine Small	NA
rs4686301	chr3	8756569	8757043	Open chromatin	Fetal Intestine Small	NA
rs4686301	chr3	8756569	8757043	Open chromatin	Fetal Muscle Leg	NA
rs4686301	chr3	8756569	8757043	Open chromatin	Fetal Muscle Leg	NA
rs4686301	chr3	8756569	8757043	Open chromatin	Fetal Muscle Trunk	NA
rs4686301	chr3	8756569	8757043	Open chromatin	Fetal Muscle Trunk	NA
rs4686301	chr3	8756569	8757043	Open chromatin	Fetal Stomach	NA
rs4686301	chr3	8756569	8757043	Open chromatin	Fetal Stomach	NA
rs4686301	chr3	8756569	8757043	Open chromatin	Fetal Thymus	NA
rs4686301	chr3	8756569	8757043	Open chromatin	Fetal Thymus	NA
rs4686301	chr3	8756569	8757043	Open chromatin	Gastric	NA
rs4686301	chr3	8756569	8757043	Open chromatin	Gastric	NA
rs4686301	chr3	8756569	8757043	Open chromatin	GM12878	INACTIVE
rs4686301	chr3	8756569	8757043	Open chromatin	H1-mesenchymal	NA
rs4686301	chr3	8756569	8757043	Open chromatin	H1-mesenchymal	NA
rs4686301	chr3	8756569	8757043	Open chromatin	H1-neuronal progenitor	NA
rs4686301	chr3	8756569	8757043	Open chromatin	H1-neuronal progenitor	NA
rs4686301	chr3	8756569	8757043	Open chromatin	H1-trophoblast	NA
rs4686301	chr3	8756569	8757043	Open chromatin	H1-trophoblast	NA
rs4686301	chr3	8756569	8757043	Open chromatin	H1ESC	INACTIVE
rs4686301	chr3	8756569	8757043	Open chromatin	H9	NA
rs4686301	chr3	8756569	8757043	Open chromatin	H9	NA
rs4686301	chr3	8756569	8757043	Open chromatin	HeLa-S3	INACTIVE

rs4686301	chr3	8756569	8757043	Open chromatin	HepG2	INACTIVE
rs4686301	chr3	8756569	8757043	Open chromatin	HMEC	INACTIVE
rs4686301	chr3	8756569	8757043	Open chromatin	HSMM	INACTIVE
rs4686301	chr3	8756569	8757043	Open chromatin	HSMMtube	ACTIVE
rs4686301	chr3	8756569	8757043	Open chromatin	HUVEC	INACTIVE
rs4686301	chr3	8756569	8757043	Open chromatin	HUVEC prol (CB)	NA
rs4686301	chr3	8756569	8757043	Open chromatin	IMR90	INACTIVE
rs4686301	chr3	8756569	8757043	Open chromatin	iPS-20b	NA
rs4686301	chr3	8756569	8757043	Open chromatin	iPS DF 19.11	NA
rs4686301	chr3	8756569	8757043	Open chromatin	iPS DF 19.11	NA
rs4686301	chr3	8756569	8757043	Open chromatin	iPS DF 6.9	NA
rs4686301	chr3	8756569	8757043	Open chromatin	iPS DF 6.9	NA
rs4686301	chr3	8756569	8757043	Open chromatin	K562	REPRESSED
rs4686301	chr3	8756569	8757043	Open chromatin	Left Ventricle	NA
rs4686301	chr3	8756569	8757043	Open chromatin	Left Ventricle	NA
rs4686301	chr3	8756569	8757043	Open chromatin	Lung	NA
rs4686301	chr3	8756569	8757043	Open chromatin	Lung	NA
rs4686301	chr3	8756569	8757043	Open chromatin	M0 macrophage (CB)	NA
rs4686301	chr3	8756569	8757043	Open chromatin	M0 macrophage (VB)	NA
rs4686301	chr3	8756569	8757043	Open chromatin	M1 macrophage (CB)	NA
rs4686301	chr3	8756569	8757043	Open chromatin	M1 macrophage (VB)	NA
rs4686301	chr3	8756569	8757043	Open chromatin	M2 macrophage (CB)	NA
rs4686301	chr3	8756569	8757043	Open chromatin	M2 macrophage (VB)	NA
rs4686301	chr3	8756569	8757043	Open chromatin	Monocytes-CD14+	INACTIVE
rs4686301	chr3	8756569	8757043	Open chromatin	Monocytes-CD14+ (PB) Roadmap	NA
rs4686301	chr3	8756569	8757043	Open chromatin	Monocytes-CD14+ (PB) Roadmap	NA
rs4686301	chr3	8756569	8757043	Open chromatin	MSC (VB)	NA
rs4686301	chr3	8756569	8757043	Open chromatin	naive B cell (VB)	NA
rs4686301	chr3	8756569	8757043	Open chromatin	Natural Killer cells (PB)	NA
rs4686301	chr3	8756569	8757043	Open chromatin	Natural Killer cells (PB)	NA
rs4686301	chr3	8756569	8757043	Open chromatin	neutrophil (CB)	NA
rs4686301	chr3	8756569	8757043	Open chromatin	neutrophil myelocyte (BM)	NA
rs4686301	chr3	8756569	8757043	Open chromatin	neutrophil (VB)	NA
rs4686301	chr3	8756569	8757043	Open chromatin	NH-A	INACTIVE
rs4686301	chr3	8756569	8757043	Open chromatin	NHDF-AD	ACTIVE
rs4686301	chr3	8756569	8757043	Open chromatin	NHEK	INACTIVE
rs4686301	chr3	8756569	8757043	Open chromatin	NHLF	INACTIVE
rs4686301	chr3	8756569	8757043	Open chromatin	Osteobl	REPRESSED
rs4686301	chr3	8756569	8757043	Open chromatin	Ovary	NA
rs4686301	chr3	8756569	8757043	Open chromatin	Ovary	NA
rs4686301	chr3	8756569	8757043	Open chromatin	Pancreas	NA
rs4686301	chr3	8756569	8757043	Open chromatin	Pancreas	NA
rs4686301	chr3	8756569	8757043	Open chromatin	Placenta	NA
rs4686301	chr3	8756569	8757043	Open chromatin	Placenta	NA
rs4686301	chr3	8756569	8757043	Open chromatin	Psoas Muscle	NA
rs4686301	chr3	8756569	8757043	Open chromatin	Right Atrium	NA
rs4686301	chr3	8756569	8757043	Open chromatin	Right Atrium	NA
rs4686301	chr3	8756569	8757043	Open chromatin	Small Intestine	NA
rs4686301	chr3	8756569	8757043	Open chromatin	Small Intestine	NA
rs4686301	chr3	8756569	8757043	Open chromatin	Spleen	NA
rs4686301	chr3	8756569	8757043	Open chromatin	Spleen	NA
rs4686301	chr3	8756569	8757043	Open chromatin	T cells (PB) Roadmap	NA
rs4686301	chr3	8756569	8757043	Open chromatin	T cells (PB) Roadmap	NA
rs4686301	chr3	8756569	8757043	Open chromatin	Thymus	NA
rs4686301	chr3	8756569	8757043	Open chromatin	Thymus	NA
