# Supplementary material for: Influence of oxytocin receptor single nucleotide sequence variants on contractility of human myometrium: an in vitro functional study
Source: BMC Med Genet. 2019 Nov 12;20:178. doi: 10.1186/s12881-019-0894-8 (PMC6852767; doi:10.1186/s12881-019-0894-8)

LD (D') Matrix results in EUR (including CEU, TSI, FIN, GBR and IBS populations) calculated with Ldlink (https://ldlink.nci.nih.gov/)

RS_number	rs1042778	rs11706648	rs237888	rs4686301	rs53576	rs237895	rs237902	rs4686302
rs1042778	1	0.377	0.912	0.343	0.489	0.306	0.234	0.197
rs11706648	0.377	1	1	0.967	0.513	0.236	0.1	0.308
rs237888	0.912	1	1	1	0.281	0.208	0.108	0.577
rs4686301	0.343	0.967	1	1	0.531	0.254	0.137	0.296
rs53576	0.489	0.513	0.281	0.531	1	0.962	0.939	0.928
rs237895	0.306	0.236	0.208	0.254	0.962	1	0.978	1
rs237902	0.234	0.1	0.108	0.137	0.939	0.978	1	1
rs4686302	0.197	0.308	0.577	0.296	0.928	1	1	1

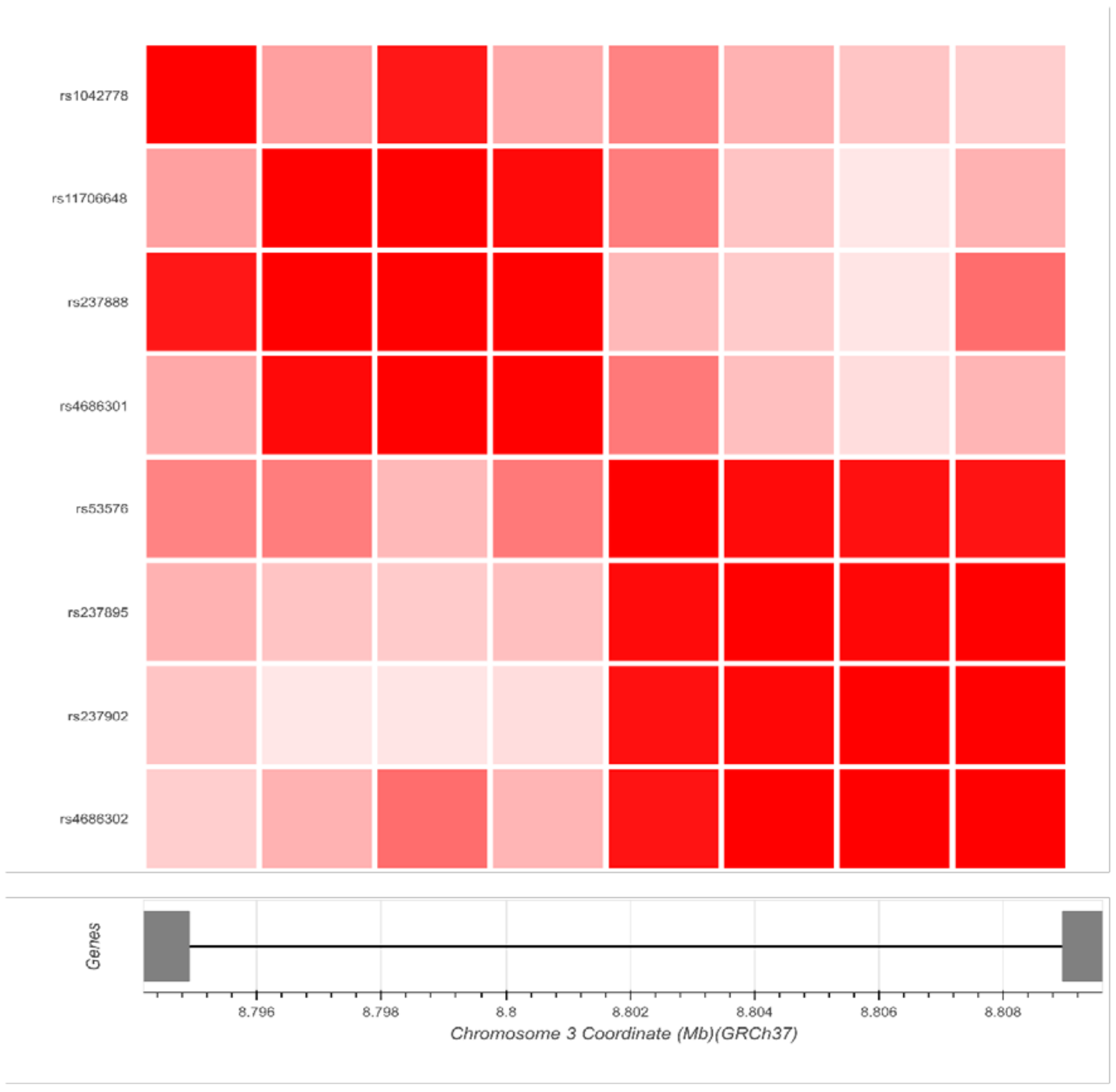

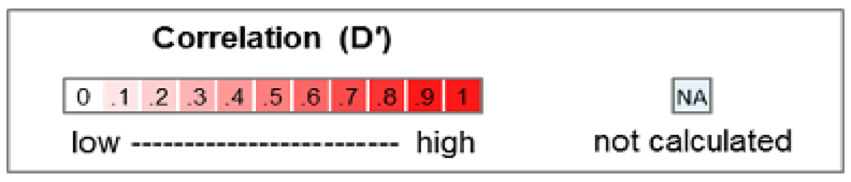

LD (R2) Matrix results in EUR (including CEU, TSI, FIN, GBR and IBS populations) calculated with Ldlink (https://ldlink.nci.nih.gov/)

RS_number	rs1042778	rs11706648	rs237888	rs4686301	rs53576	rs237895	rs237902	rs4686302
**rs1042778**	1	0.111	0.032	0.087	0.078	0.039	0.044	0.003
**rs11706648**	0.111	1	0.03	0.884	0.067	0.018	0.01	0.027
**rs237888**	0.032	0.03	1	0.028	0.009	0.004	0.002	0.003
**rs4686301**	0.087	0.884	0.028	1	0.068	0.02	0.017	0.026
**rs53576**	0.078	0.067	0.009	0.068	1	0.718	0.232	0.062
**rs237895**	0.039	0.018	0.004	0.02	0.718	1	0.324	0.093
**rs237902**	0.044	0.01	0.002	0.017	0.232	0.324	1	0.065
**rs4686302**	0.003	0.027	0.003	0.026	0.062	0.093	0.065	1

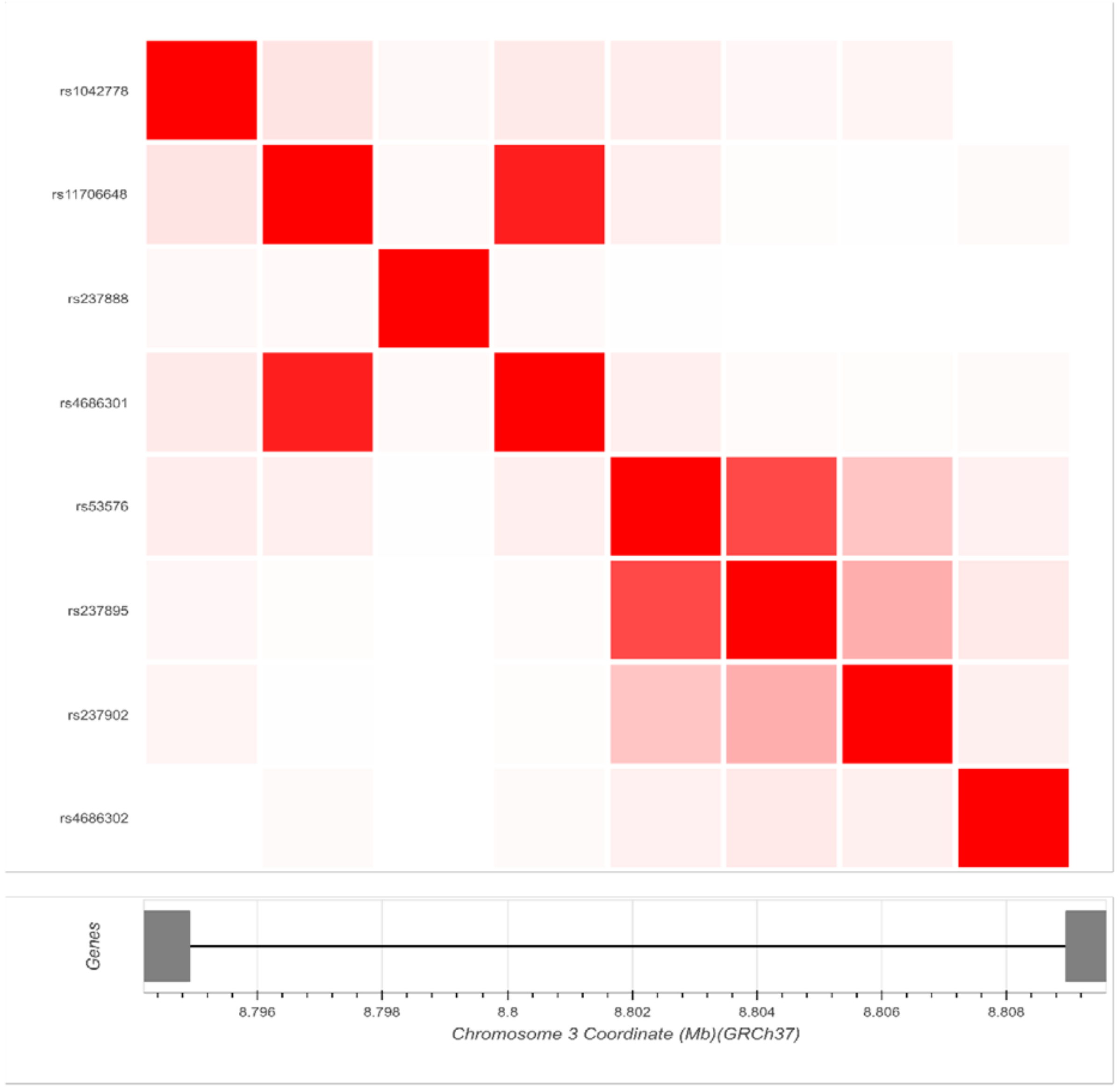

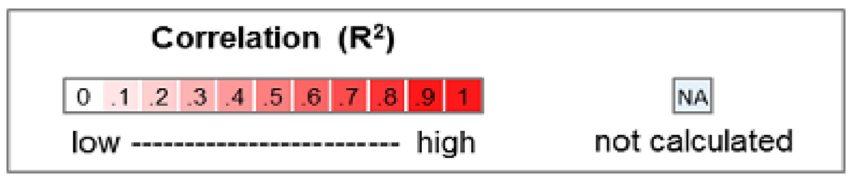

Supplement: Supplementary file 2 — Additional file 2: Table S2. Link disequilibrium values of the eight common OXTR sequence variants previously shown to be associated with a relevant perinatal outcome as determined using LDlink 3.0. [file 12881_2019_894_MOESM2_ESM.pdf]
