# Supplementary material for: Influence of oxytocin receptor single nucleotide sequence variants on contractility of human myometrium: an in vitro functional study
Source: BMC Med Genet. 2019 Nov 12;20:178. doi: 10.1186/s12881-019-0894-8 (PMC6852767; doi:10.1186/s12881-019-0894-8)
Supplement: Supplementary file 3 — Additional file 3: Table S3. Distribution of the various sequence variant alleles in the study population. [file 12881_2019_894_MOESM3_ESM.pdf]

Table S3. Distribution of the various sequence variant alleles in the study population.

Variant	Genotype	n (%)	Hardy-Weinberg Equilibrium (p-value)
rs1042778 (G/T)	GG	21 (35.0)	1.00
	GT	29 (48.3)	
	TT	10 (16.7)	
rs11706648 (A/C)	AA	29 (48.3)	0.13
	AC	28 (46.7)	
	CC	3 (5.0)	
rs237888 (T/C)	TT	54 (91.5)	**0.003**[a]
	CT	4 (6.8)	
	CC	1 (1.7)	
rs4686301 (C/T)	CC	32 (53.33)	0.38
	CT	25 (41.7)	
	TT	3 (5.0)	
rs53576 (G/A)	GG	25 (41.7)	0.46
	AG	29 (48.3)	
	AA	6 (10.0)	
rs237895 (C/T)	CC	18 (30.0)	0.10
	CT	34 (56.7)	
	TT	8 (13.3)	
rs237902 (G/A)	GG	26 (43.3)	0.12
	AG	30 (50.0)	
	AA	4 (6.7)	
rs4686302 (C/T)	CC	45 (75.0)	0.15
	CT	15 (25.0)	
	TT	0 (0.0)	

Hardy-Weinberg Equilibrium was tested; [a] indicating disequilibrium.
