# Supplementary material for: Influence of oxytocin receptor single nucleotide sequence variants on contractility of human myometrium: an in vitro functional study
Source: BMC Med Genet. 2019 Nov 12;20:178. doi: 10.1186/s12881-019-0894-8 (PMC6852767; doi:10.1186/s12881-019-0894-8)
Supplement: Supplementary file 4 — Additional file 4: Table S4. OXTR-mRNA in the various reference and variant allele groups. [file 12881_2019_894_MOESM4_ESM.pdf]

Table S4. *OXTR*-mRNA[a] in the various reference and variant allele groups.

	OXTR-mRNA (CNRQ)				
	Reference group		Variant group		p-value[b]
	n/N	Mean ± SD	n/N	Mean ± SD	
rs1042778	21/60	1.14 ± 0.62	39/60	1.27 ± 0.84	0.540
rs11706648	29/60	1.13 ± 0.62	31/60	1.31 ± 0.88	0.349
rs237888	54/59	1.24 ± 0.77	5/59	1.22 ± 0.76	0.962
rs4686301	32/60	1.09 ± 0.64	28/60	1.38 ± 0.88	0.143
rs53576	25/60	1.14 ± 0.67	35/60	1.28 ± 0.83	0.479
rs237895	18/60	1.18 ± 0.74	42/60	1.24 ± 0.79	0.786
rs237902	26/60	1.22 ± 0.68	34/60	1.23 ± 0.84	0.961
rs4686302	45/60	1.22 ± 0.78	15/60	1.23 ± 0.74	0.943

[a] *OXTR*-mRNA levels were normalized to *GAPDH*-mRNA, 18S rRNA and *EEF1A1*-mRNA and are expressed as CNRQ (see Methods).
[b] For each sequence variant, the p-value represents the statistical significance of the difference between reference and variant groups as calculated using linear regression.
n, number of samples either in the reference group or in the variant allele group of a given variant
N, number of samples for which data on a given variant are available
SD, standard deviation
